# Results from rapid-cycle recurrent genomic selection in spring bread wheat

**DOI:** 10.1093/g3journal/jkad025

**Published:** 2023-01-26

**Authors:** Susanne Dreisigacker, Paulino Pérez-Rodríguez, Leonardo Crespo-Herrera, Alison R Bentley, José Crossa

**Affiliations:** International Maize and Wheat Improvement Center (CIMMYT), Km 45 Carretera México-Veracruz, Texcoco, Edo. de México, CP 56100, México; Colegio de Postgraduados, Montecillos, Edo. de México, CP 56264, México; International Maize and Wheat Improvement Center (CIMMYT), Km 45 Carretera México-Veracruz, Texcoco, Edo. de México, CP 56100, México; International Maize and Wheat Improvement Center (CIMMYT), Km 45 Carretera México-Veracruz, Texcoco, Edo. de México, CP 56100, México; International Maize and Wheat Improvement Center (CIMMYT), Km 45 Carretera México-Veracruz, Texcoco, Edo. de México, CP 56100, México; Colegio de Postgraduados, Montecillos, Edo. de México, CP 56264, México

**Keywords:** genomic-assisted breeding, molecular markers, pedigree information, rapid-cycle recurrent genomic selection, wheat, genomic prediction, GenPred, shared data resources

## Abstract

Genomic selection (GS) in wheat breeding programs is of great interest for predicting the genotypic values of individuals, where both additive and nonadditive effects determine the final breeding value of lines. While several simulation studies have shown the efficiency of rapid-cycling GS strategies for parental selection or population improvement, their practical implementations are still lacking in wheat and other crops. In this study, we demonstrate the potential of rapid-cycle recurrent GS (RCRGS) to increase genetic gain for grain yield (GY) in wheat. Our results showed a consistent realized genetic gain for GY after 3 cycles of recombination (C_1_, C_2_, and C_3_) of bi-parental F_1_s, when summarized across 2 years of phenotyping. For both evaluation years combined, genetic gain through RCRGS reached 12.3% from cycle C_0_ to C_3_ and realized gain was 0.28 ton ha^−1^ per cycle with a GY from C_0_ (6.88 ton ha^−1^) to C_3_ (7.73 ton ha^−1^). RCRGS was also associated with some changes in important agronomic traits that were measured (days to heading, days to maturity, and plant height) but not selected for. To account for these changes, we recommend implementing GS together with multi-trait prediction models.

## Introduction

The widespread adoption of genomic selection (GS) in plant and animal breeding has strongly been driven by new sequencing technologies that generate abundant and inexpensive molecular markers (Meuwissen *et al.* 2001; Bernardo and Yu 2007; Lorenzana and Bernardo 2009). GS significantly increases prediction accuracy over marker-assisted selection for low heritability traits (de los Campos *et al.* 2009, 2010, 2012; Crossa *et al.* 2010, 2011, 2013, 2014, 2017; González-Camacho *et al.* 2012, 2016; Heslot *et al.* 2012, 2014; Pérez-Rodríguez *et al.* 2012; Riedelsheimer *et al.* 2012; Windhausen *et al.* 2012; Zhao *et al.* 2012; Hickey *et al.* 2014; Dreisigacker *et al.* 2021). GS involves predicting breeding values that comprise the parental average (half the sum of the breeding values of both parents) and a deviation of Mendelian sampling. GS can therefore be applied in 2 different contexts: (1) predicting additive effects in early generations of a breeding program such that a rapid selection cycle with a short breeding interval (i.e. GS at the F_2_ level of a bi-parental cross) is achieved (Crossa *et al.* 2014) and (2) predicting the genotypic values of individuals where both additive and nonadditive effects determine the final commercial value of the lines (i.e. lines established in a sparse multi-environment field evaluation). Gaynor *et al.* (2017) clearly suggested separating the use of GS for parental selection or population improvement for crosses based on breeding values from product development that consists of testing lines and deriving varieties based on total genetic values.

Gholami *et al.* (2021) emphasized that plant breeders traditionally focus on product development, rather than identifying parents for new crosses. In other words, plant breeders have been more inclined to use total genetic values comprising the complete genetic contribution to the phenotype than the additive genetic value necessary for line improvement and crossing of new parental lines.

The International Maize and Wheat Improvement Center (CIMMYT, https://www.cimmyt.org) has explored GS as a new applied breeding tool since 2009 (de los Campos *et al.* 2009; Crossa *et al.* 2010, 2019, 2021; Dreisigacker *et al.* 2021). Genomic estimated breeding values (GEBVs) are routinely implemented and used as a decision tool by breeders. Studies at CIMMYT have evaluated using GEBVs for germplasm that have not been included in trials, for applying GS in early selection to shorten cycle time, and for using sparse testing (Atanda *et al.* 2022). CIMMYT has also built the basis for a more informed screening of novel allelic diversity in germplasm collections by genotyping a substantial part of the available accessions from its gene banks (Sansaloni *et al.* 2020; Martini *et al.* 2021). Extensive studies utilizing the GEBVs of traits from wheat germplasm bank accessions (Crossa *et al.* 2016) were performed to explore its potential for harnessing genetic resources (Gholami *et al.* 2021; Martini *et al.* 2021). The practical application of GS has been studied and applied based on the individual breeder’s decision. However, most recently, there has been a clear focus on shortening the breeding cycle by advancing and selecting lines quickly up to the F_4_ and F_5_ generations, sparse testing these lines at several locations (some belonging to the target population of environments already defined), and recycling them based on total genetic values.

The CIMMYT Global Maize Program has been highly successful in achieving important genetic gains in bi-parental populations in drought environments (Beyene *et al.* 2015, 2019). Gains were achieved from significant decreases in the breeding cycle, and just as importantly, hybrids from lines developed using GS have proved to be productive, high yielding, and stable across several drought and optimal environments. The achievements reported by Beyene *et al.* (2015, 2019) concluded that genetic gain in maize hybrids developed with GS was remarkable, considering the commercial checks used in the studies were the best in the multi-environment trials. Beyene *et al.* (2015) concluded that “the average gain observed under drought in our study using GS was two- to four folds higher than what has been reported from conventional phenotypic selection”. Moreover, Zhang *et al.* (2017) designed rapid-cycle recurrent GS (RCRGS) of multi-parental crosses with important significant gains per cycle in tropical maize in Mexico.

The CIMMYT Global Wheat Program started implementing GS as a routine breeding tool in 2013, and, since then, has made important contributions by developing and testing several new genome-enabled prediction models (Dreisigacker *et al.* 2021) including G × E interaction and multi-trait, multi-environment genome-based predictions. For decades, CIMMYT wheat breeders have been using a standard pedigree system for crosses, which makes it possible to accurately predict breeding values based on the additive relationship matrix (***A***) and its incorporation in the statistical analyses of multi-environment trials by modeling G × E interaction with the factor analytic model as shown in Crossa *et al.* (2006) and Burgueño *et al.* (2007). These authors concluded that epistatic interaction in wheat is important, and it is necessary to correctly assess additive, additive × additive, additive × environment, and additive × additive × environment interactions in wheat breeding.

Pérez-Rodríguez *et al.* (2012) assessed the predictive ability of linear and nonlinear models on the marker effects using high-density genotypic data in wheat. The linear models were Bayesian LASSO, Bayesian ridge regression, Bayes A, and Bayes B, whereas the nonlinear models were the reproduced kernel Hilbert space (RKHS) regression, Bayesian regularized neural networks (BRNN), and radial basis function neural networks (RBFNN). It was found that the 3 nonlinear models had consistently better prediction accuracy than the linear regression specification. Pérez-Rodríguez *et al.* (2012) concluded the importance of epistasis in wheat and coincided with the results of Crossa *et al.* (2006) and Burgueño *et al.* (2007) using the additive relationship matrix ***A***. The results also agreed with Gianola *et al.* (2006), Long *et al.* (2010), and González-Camacho *et al.* (2012), which concluded that nonparametric treatment of markers may account for epistatic effects not captured by linear additive regression models and seemed to be useful for predicting quantitative traits with different complex underlying gene action under varying types of interaction in different environmental conditions.

Early GS studies utilizing CIMMYT wheat datasets have already shown that molecular markers increased genome-wide prediction abilities over the pedigree-derived models (de los Campos *et al.* 2009; Crossa *et al.* 2010). Furthermore, when molecular markers and pedigree information are jointly considered, the prediction abilities are slightly, but consistently, superior to the marker or pedigree-derived models on their own. The CIMMYT Global Wheat Program has not yet applied GS at early breeding stages for population improvement. Nevertheless, as early as 2009, an extensive proof-of-concept experiment was established with the objective of incorporating genomic predictions for increased grain yield in an early breeding generation (Bonnett *et al.* 2022) to compare the realized response to selection based on 3 prediction models. Experiment 2 in the study of Bonnett *et al.* (2022) compared the predictive ability of the different GEBV calculation methods in F_2_ using a set of single plant-derived F_2:4_ lines from randomly selected F_2_ plants. Results showed a significant positive correlation between the observed yield of F_2:4_ lines and the predicted yield GEBVs of F_2_ single plants based on the nonlinear RKHS method. For the first time in wheat, results showed the potential for the application of GS in early generations of wheat breeding and the importance of using the appropriate statistical model for GEBVs calculation.

Based on the initial results of Bonnett *et al.* (2022), a second RCRGS experiment was established. The main objective of this study was to perform 3 genomic-assisted recurrent selection cycles in the greenhouse based on a training population of F_4_ lines and to estimate realized genetic gains for grain yield in each cycle and across cycles.

## Materials and methods

### Developing the training population (C_0_)

The training population (C_0_) consisted of 1,609 F_4_ lines derived from 14 F_2_ families, which were based on 16 parents from the CIMMYT spring bread wheat breeding program. Eleven F_2_ families comprised 94 to 95 F_4_ lines and 3 F_2_ families included 186 to 190 lines. F_2_ individuals were genotyped with the Infinium iSelect 90K SNP genotyping array (Wang *et al.* 2014) and genotype calling was performed using GenomeStudio Software v2011.1 (https://www.illumina.com). The F_2_ individuals were phenotyped as F_4_ lines at the Campo Experimental Norman E. Borlaug (CENEB) in Ciudad Obregón, northern Mexico. The F_4_ trial was sown as an augmented block design with 2 replications, including the line “BORLAUG100 F2014” as a repeated check. The phenotypic data included grain yield (GY, ton ha^−1^), days to heading (DTH, days), and plant height (PH, cm). Agronomic traits (DTH and PH) were only measured in one replication. Best linear unbiased estimators (BLUEs) for GY were assessed for all genotypes. A numerical relationship matrix (***A***) derived from the pedigree was also available for all individuals in the training set. This relationship matrix was computed by the “coefficient of parentage (COP)” using the BROWSE software (McLaren *et al.* 2000, 2005).

### Cycle 0 phenotypic selection and formation of cycle 1

**Fig. 1.**
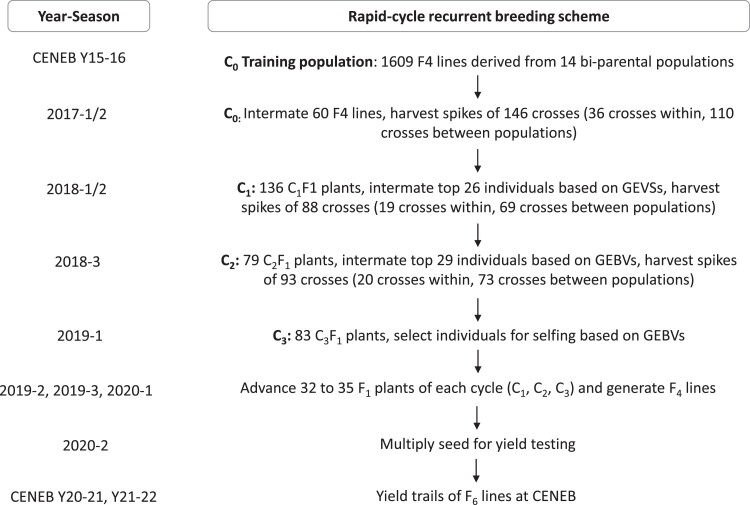
Rapid-cycle recurrent breeding scheme starting with the formation of the training population to performing final yield trials.

In cycle 0 (C_0_), the 10 highest yielding lines of 6 F_4_ families each were selected as parents to form cycle 1 (C_1_). The 6 F_4_ families were selected based on several criteria: their rank in GY (BLUEs) in the training population, GY heritability, and the estimated genomic prediction ability within and between F_4_ families. The agronomic traits (DTH and PH) were not considered when making selections. The 60 selected F_4_ lines were planted in the greenhouse on 3 different dates (3 pots with 6–7 plants per F_4_ line at each date). C_1_ was formed by intermating the F_4_s (Fig. 1). Six crosses were performed within each selected F_4_ family (36 crosses) and 10 crosses between 11 pairs of F_4_ families (110 crosses), which were chosen based on the average genomic prediction ability between them. Each F_4_ family was used in intercrosses at least 3 times. The F_1_ seed of each cross was harvested and threshed to form C_1_.

### Recombination using GS in cycle 1, cycle 2, and cycle 3

In C_1_, 136 F_1_s were planted at 2 different dates in the greenhouse (1 pot with 3 plants per F_1_ at each date). DNA was extracted from bulked tissue and shipped to TraitGenetics GmbH, Germany, for genotyping with the Illumina 20K microarray (https://www.traitgenetics.com). GEBVs were calculated for all 136 F_1_s. The top 26 C_1_F_1_s were selected and intermated to form the cycle 2 (C_2_) population. Like C_1_, crosses were performed within and between families (19 and 69 crosses, respectively), C_2_F_1_ seed of each cross was harvested and threshed. In C_2_, the recombination protocol was repeated. The top 29 C_2_F_1_s were intermated to form cycle 3 (C_3_). The number of F_1_s planted per cycle, the number of parents selected for next cycle recombination, and the number of crosses performed are shown in Fig. 1. After C_2_ recombination, C_3_F_1_ were genotyped and GEBVs calculated, but they were not recombined.

### Line development and phenotypic evaluation of the selection cycles

After recurrent selection, 32 to 35 F_1_s from each cycle (C_1_ to C_3_) were selfed to derive F_6_ lines. Not all F_1_s within a selection cycle had sufficient seed for selfing. Therefore, a distributed set of F_1_s was chosen. The GEBVs of the selfed F_1_s ranked between 1 and 58 in each cycle, and about 50% of F_1_s were used in recombination, while the residual F_1_s were not crossed (Supplementary Table 1). F_1_s were advanced to the F_4_ breeding generation in the greenhouse via “selected bulks.” Residual F_1_ seed was planted in trays in the greenhouse, and several visually “good” spikes were bagged to derive F_2_ seed. This advancement procedure was repeated up to the F_4_ generation. F_5_ seed was harvested and planted for seed multiplication at CIMMYT in El Batan, Mexico, in 0.5 m plots; plant off-types were eliminated.

A total of 118 lines were field tested together with the cv. “BORLAUG100 F2014” as standard check for 2 crop cycles (2020–2021 and 2021–2022) at CENEB. The lines were grown in 2 trials of 60 entries (59 lines + check) in an incomplete block design with 2 replications. The evaluations were conducted under optimal conditions, i.e. 500 mm of irrigation water, mechanized and chemical control of weeds, diseases, and pests. The 240 plots were of dimension 2.8 × 1.6 m and sown at a seed rate of 120 kg ha^−1^. The same agronomic traits as in the training population were measured, GY, DTH, and PH, in addition days to maturity (DTM). Lines included 101 F_6_ lines derived from the recurrent GS cycles and 17 of the 60 C_0_ parents and the check. Means of GY BLUEs of each recurrent selection cycle were compared, and differences were determined using the least significant difference (LSD at 5% significance). The heritability of the trials was estimated from the variance components using the equation:


$$H^{2}=\frac{\sigma_{g}^{2}}{\sigma_{g\;}^{2}+\;\frac{\sigma_{ge}^{2}}{e}+\;\frac{\sigma^{2}}{re}}$$

with*r* = number of replications, *e* = number of environments (years), σ^2^ = error variance, $\sigma_{g\;}^{2}$ = genotypic variance, and $\sigma_{ge\;}^{2}$ = G × Y variance.

### SNP genotyping and filters

As described above, the initial training population was genotyped with the Infinium iSelect 90 K SNP genotyping array for wheat and the recurrent selection F_1_ plants with the lower-density Illumina Infinium 20 K wheat SNP array. A total of 7,815 markers overlapped between platforms. We imputed missing marker genotypes at random according to allele frequencies and subsequently removed monomorphic markers and markers with a minor allele frequency smaller than 0.05. After this quality control, 7,139 markers were available for further analysis. The 101 derived F_6_ lines were also genotyped with the Illumina Infinitum array (TraitGenetics) to compute the genetic diversity maintained in each selection cycle.

### Genomic prediction models

We considered 4 different prediction models to fit the training population for selecting the best parents to be crossed and initiate the RCRGS as well as for selecting the best F_1_s in each recurrent cycle: (1) the genomic best linear unbiased prediction model (GBLUP), (2) GBLUP including the pedigree information (P + GBLUP), (3) Reproducing Kernel Hilbert Spaces with Kernel Averaging (RKHS-KA) method, and (4) Reproducing Kernel Hilbert Spaces with Kernel Averaging and Pedigree (P + RKHS-KA). While all models were computed, selections during the RCRGS scheme were based only on Model 4.

For the prediction (GEBV) and selection of the best parental candidates for the next cycle, we focused on (1) assessing additive effects by including the pedigree (P) information (numerator relationship matrix) and thus emphasizing between family variance, and (2) including genotypic values of individuals where both additive and nonadditive are included on the genomic information (RKHS-KA). Using pedigree and marker information together has been successful in decreasing the interval cycle at the early stages of population improvement (Crossa *et al.* 2017; Bonnet et al. 2022).

#### GBLUP model

The GBLUP model has become widely used in genomic prediction (e.g. Endelman 2011). The model can be written as:


(1) y=μ1+u+e,

where***y*** is a vector with the response variable of dimension *n* × 1 (phenotypes), *μ* is an intercept, **1** is a vector of ones, $u∼N(0,\;\sigma_{u}^{2}K)$ corresponds to the random effect of wheat lines, $\sigma_{u}^{2}$ is the variance parameter associated to the wheat lines, and ***K*** = ***MM***^′^**/***p* is a genomic relationship matrix derived from markers (e.g. Lopez-Cruz *et al.* 2015) with ***M*** the matrix of markers centered and standardized by columns and *p* the number of markers, $e∼N(0,\;\sigma_{e}^{2}I)$ the vector of random error terms, with $\sigma_{e}^{2}$ the variance parameter associated to the error, and ***I*** denotes the identity matrix.

#### The pedigree + molecular marker model (P + GBLUP)

The pedigree + molecular marker model takes into account the pedigree information of the wheat lines represented by the numerical relationship matrix (***A***) and the genomic relationship matrix derived from markers described before (Eq. 1). The full genetic model is:


(2) y=μ1+a+u+e,

where$a∼N(0,\sigma_{a}^{2}A)$ is the vector of additive random effects for wheat lines whose variance–covariance matrix is obtained from the numerator relationship matrix (***A***) derived from the coefficient of co-ancestry between the wheat lines and $\sigma_{a}^{2}$ is a variance parameter associated with the additive relationship matrix derived from pedigree information and the rest of the terms has been described before.

#### Reproducing kernel Hilbert spaces with kernel averaging

The Gaussian kernel commonly used in genomic prediction is $K(x_{i},x_{i^{\prime }})=\exp\;(-hd_{ii^{\prime }}^{2})$ (e.g. Pérez-Rodríguez *et al.* 2012), where $d_{ii^{\prime }}$ is the distance based on markers between individuals *i*, *i*^′^ (*i* = 1, …, *n*), the bandwidth parameter controls how fast the covariance function drops as a function of the distance. The estimation of the bandwidth parameter is computationally demanding and to overcome this problem, de los Campos *et al.* (2010) proposed to fit a model that includes several kernels, each one with its own bandwidth parameter. The RKHS-KA with 3 kernels is given by:


(3) $$y=\mu 1+u_{1}+u_{2}+u_{3}+e,$$

where$u_{1}∼N(0,\;\sigma_{u1}^{2}K_{1}),\;u_{2}∼N(0,\;\sigma_{u2}^{2}K_{2}),\;\;u_{3}∼N(0,\;\sigma_{u3}^{2}K_{3})$ and ***e*** distributed independently, with 3 different Gaussian kernels computed with bandwidth parameters $h_{1}=\frac{1}{5m},\;$$h_{2}=\frac{1}{m}$, $h_{3}=\frac{5}{m}$, with *m* that corresponds to the median squared Euclidean distances between lines without including the diagonal entries (Pérez-Rodríguez and de los Campos 2014), $\sigma_{u1}^{2},\;\sigma_{u2}^{2}$, $\sigma_{u3}^{2}$ corresponds to variance parameters associated to ***u***_1_, ***u***_2_, ***u***_3_ respectively. The rest of the terms has been already described.

#### P + RKHS-KA model

This model is an extension of model (3) where we include a random effect to take the additive relationship matrix derived from pedigree into account, the model is given by:


(4) $$y=\mu 1+a+u_{1}+u_{2}+u_{3}+e,$$

whereall terms have been described previously and $a∼N(0,\sigma_{a}^{2}A)$ distributed independently from ***u***_1_, ***u***_2_, ***u***_3_, and ***e***.

Models (1)–(4) were fitted using the “BGLR” statistical package (Pérez-Rodríguez and de los Campos 2014) using the R Software (R Core Team 2018).

### Assessing the genetic diversity in each selection cycles

Based on the genomic data, we computed Nei’s standard genetic distance D (Nei 1972) between the 60 parents in C_0_ and the phenotypically evaluated F_6_ lines from the different selection cycles C_1_, C_2_, and C_3_. Principal component analysis (PCA) was performed to assess the genetic relationship between lines. The matrix of genetic distances and PCA were generated with the packages “adegenet” and “ggplot2” using the R Software (R Core Team 2018).

## Results

### Variation for heritability and prediction ability of GY differs between families in the training population

The average GY of the evaluated F_4_ families in the training population ranged from 5.91 to 7.23 ton ha^−1^ (Table 1). Low-to-high GY heritability was observed in the F_4_ families, with an *h^2^* of 0.01 for family 8 (WAXBI/3/ATTILA*2/PBW65*2//MURGA) to an *h^2^* of 0.73 for family 4 (NELOKI//KACHU/KIRITATI). To select the parents for rapid cycle recombination, we implemented random cross-validation within and between the F_4_ families. Prediction abilities using the P + RKHS-KA model within the families were significantly higher than between the families but varied widely. The highest prediction ability within families was 0.496 for family 12 (MUTUS*2/JUCHI/6/COPIO) and, between families, it was 0.310 for family 3 (NELOKI//KFA/2*KACHU). We further assessed the COP between families. Mean COPs had an overall lower range compared with prediction abilities, the most distinct family being family 2 (NELOKI/WAXBI) with a value of 0.483 (Table 1). Out of the 14 F_4_ families, we selected 6 families (families 2, 3, 4, 11, 12, and 13) with the overall highest GY heritability and good prediction ability within and between families. The 10 highest-yielding F_4_ lines of each family were selected as parents in C_0_ (Supplementary Table 2). The average GY of the 10 F_4_ lines per family varied, with 2 families each showing higher, medium, and lower GY when compared to all families.

**Table 1.** Summary statistics of 14 F_4_ families including 1,609 F_4_ lines in the training population.

No.	Cross/family name	No. F_4_ lines	Mean—highest yielding 10 F_4_ lines			Mean—all F4 lines				Prediction within families	Prediction between families	COP between families
			GY*^a^*	PH	DTH	GY	$\sigma_{g}^{2}$	$\sigma_{e}^{2}$	*h^b^*			
1	PAURAQ/3/ATTILA*2/PBW65*2//W485/HD29	94	7.45	101.1	72.9	6.94	0.085	0.132	0.39	0.005	−0.028	0.353
2	**NELOKI/WAXBI*^b^***	**190**	**7**.**65**	**98**.**0**	**78**.**0**	**7**.**03**	**0**.**072**	**0**.**092**	**0**.**44**	**0**.**353**	**0**.**037**	**0**.**483**
3	NELOKI//KFA/2*KACHU	**190**	**7**.**21**	**93**.**4**	**74**.**4**	**6**.**43**	**0**.**138**	**0**.**107**	**0**.**56**	**0**.**267**	**0**.**301**	**0**.**410**
4	**NELOKI//KACHU/KIRITATI**	**95**	**7**.**04**	**94**.**4**	**76**.**3**	**5**.**91**	**0**.**354**	**0**.**134**	**0**.**73**	**0**.**338**	**0**.**067**	**0**.**413**
5	COPIO/6/MUTUS*2/AKURI	95	7.39	97.5	73.9	6.74	0.022	0.315	0.07	0.184	−0.113	0.181
6	PARUS/FRANCOLIN#1//KFA/2*KACHU	95	7.23	101.6	75.1	6.60	0.096	0.077	0.55	0.021	0.230	0.326
7	ATTILA*2/PBW65//MUU#1/3/FRANCOLIN#1/4/KACHU/KINDE	95	7.11	97.5	76.2	6.49	0.081	0.103	0.44	−0.001	0.085	0.360
8	WAXBI/3/ATTILA*2/PBW65*2//MURGA	95	7.36	95.5	77.6	6.81	0.003	0.211	0.01	0.125	0.088	0.383
9	WAXBI/4/ATTILA*2/PBW65//MUU#1/3/FRANCOLIN#1	94	7.75	95.3	73.8	7.23	0.014	0.321	0.04	0.031	0.033	0.396
10	WAXBI//KFA/2*KACHU	95	7.54	97.3	74.8	7.00	0.084	0.177	0.32	0.093	0.218	0.345
11	SUP152/BAJ#1//KFA/2*KACHU	**95**	**7**.**66**	**99**.**8**	**72**.**1**	**6**.**97**	**0**.**121**	**0**.**130**	**0**.**48**	**0**.**303**	**0**.**250**	**0**.**340**
12	MUTUS*2/JUCHI//COPIO	**95**	**7**.**26**	**95**.**5**	**78**.**1**	**6**.**64**	**0**.**137**	**0**.**112**	**0**.**55**	**0**.**496**	**0**.**059**	**0**.**168**
13	**KACHU/KINDE//SUP152**	**95**	**7**.**03**	**97**.**7**	**72**.**7**	**6**.**33**	**0**.**094**	**0**.**117**	**0**.**44**	**0**.**179**	**0**.**091**	**0**.**434**
14	KACHU/KINDE//NELOKI	186	7.63	103.0	76.4	6.75	0.063	0.160	0.28	0.010	0.098	0.401

*G*Y: Grain yield (ton ha^−1^), PH: Plant height (cm), DTH: Days to heading (days), $\sigma_{g}^{2}$: genotypic variance, $\sigma_{e}^{2}$: residual variance.

*F*rom the families marked in bold, the 10 highest yielding lines were selected as parents to from cycle C_1_.

### GS accuracies varied between set of lines

F_1_ individuals in each recombination cycle were predicted using the entire training population, which was not updated throughout the study. GEBVs calculated using the P + RKHS-KA model ranged from 6.42 to 7.50 ton ha^−1^ among F_1_s across cycles. The mean of the GEBVs constantly increased with each cycle from 6.71 ton ha^−1^ in C_0_ to 7.20 ton ha^−1^ in C_3_, with an average increase of 0.16. GEBV means between cycles were, however, not always significantly different (Supplementary Table 3). This steady increase of GEBVs was not apparent for the F_1_ individuals that were selected as parents and the individuals that were selected to be advanced to F_6_ for yield evaluation (Supplementary Tables 1–3). In both sets of selected lines, the mean GEBVs were slightly lower in C_2_ compared to C_1_ and increased again in C_3_. In recombination cycle C_2_, the smallest number of F_1_s was generated, and the selected sets of parents and individuals advanced included only 30–40% of the total number of F_1_s, which might explain this result.

In addition to the P + RKHS-KA model, which was the only model applied for the selection of parents in each recombination cycle, GEBVs using the RKHS-KA model without the numerical relationship matrix ***A*** and the standard GBLUP model with and without ***A*** were calculated to corroborate the correlation between GEBVs of different models in an RCRGS setting (Supplementary Table 3). The GEBVs of the 3 additional models showed very similar trends across the recombination cycles regarding the P + RKHS-KA model, while the significance between cycles varied. Interestingly, the GEBVs of the models without ***A*** showed lower predicted GY values and lower and nonsignificant means in cycle C_3_ compared to cycle C_1_ for the selected parents and the individuals that were advanced. The P + GBLUP model predicted the highest yields. The correlations between the GEBVs of the models were positive and ranged on average from 0.32 to 0.94. The correlations were highest in C_0_ (0.91) and declined in the subsequent cycles. Correlations also declined in the selected subsets of F_1_s in comparison to the entire F_1_ population.

### Rapid cycling recombination GS for grain yield increases realized genetic gains

**Fig. 2.**
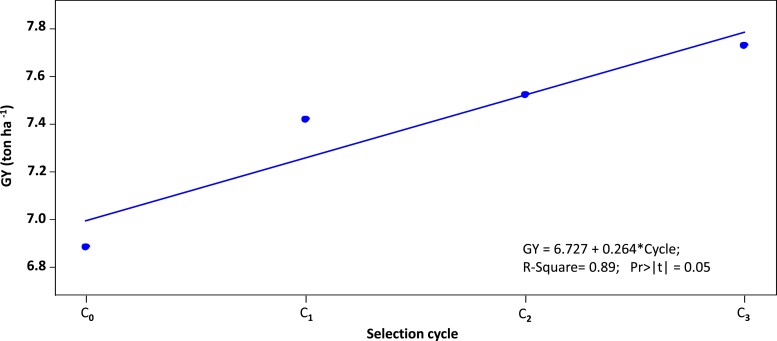
Mean GY (ton ha^−1^) for each selection cycle: C_0_, C_1_, C_2_, and C_3_.

Four groups of entries derived from C_0,_ C_1,_ C_2_, and C_3_ and the repeated check were used for field evaluation at CENEB across 2 crop cycles. The mean GY for each cycle and the average gain per cycle are shown in Fig. 2 and also presented in Table 2. Overall, GY in the trial was slightly lower in Year 1 (2020–2021), reaching 7.42 ton ha^−1^, but not significantly different from Year 2 (2021–2022) with an average of 7.51 ton ha^−1^ (Table 2). In Year 1 and over the 2 years combined, the entries of the base selection cycle C_0_, (using 17 out of the 60 initial parents) had the lowest GY, with an average of 6.88 ton ha^−1^ across years. The same 17 parents revealed an average GY of 7.31 ton ha^−1^ in the original training population, which was the same as the 60 initial parents used in C_0_ (7.31 ton ha^−1^) and higher than the average GY (6.71 ton ha^−1^) across all entries in the training population (1,609 entries). In Year 2, C_0_ entries showed a higher GY average than C_1_ and C_2_ entries (Table 2).

**Table 2.** Mean yield comparison and least significant difference (LSD) of recurrent cycles evaluated at CENEB during 2020–2021 (year-1) and 2021–2022 (year-2) growing seasons.

Cycle	No. of lines	Mean GY (ton ha^−1^)	Tukey grouping	
Year 1 (Y2020–2021)		[LSD (0.05) = 0.212]		
C_0_	17	6.11	A	
C_1_	32	7.58	B	
C_2_	34	7.60	B	
C_3_	35	7.73	B	
Year 2 (Y2021–2022)		[LSD (0.05) = 0.221]		
C_0_	17	7.65	A	B
C_1_	32	7.26	C	
C_2_	34	7.45	C	B
C_3_	35	7.73	A	
Combined		[LSD (0.05) = 0.153]		
C_0_	17	6.88	A	
C_1_	32	7.42	B	
C_2_	34	7.52	B	
C_3_	35	7.73	C	

In both years, the performance of the 35 C_3_ entries surpassed the GY of all the other cycles. The average GY among C_3_ entries was 7.73 ton ha^−1^ in both years and across years. The higher GY in C_3_ was not significant in Year 1 but in Year 2 and for both years combined.

The average gain per cycle for each year and combined across years ranged from −0.39 ton ha^−1^ to 1.47 ton ha^−1^. Across both years, the realized genetic gains were 0.28 ton ha^−1^, with the highest gains in C_1_ followed by C_3_ and C_2_.

### Unselected flowering and height traits decreased across recombination cycles

In the training population, genetic correlations of PH, DTH, and DTM with GY were in general low (−0.07, 0.11, and 0.02, respectively) and high levels of indirect selection were not expected. The effects of GS on the unselected agronomic traits PH, DTH, and DTM are presented in Supplementary Table 4. All 3 traits were only evaluated in one replication in each of the yield trials. Some plots showed segregation for one of the traits, likely since lines were derived from selected bulks. On average, lines flowered significantly earlier (81.9 and 79.3 days) and matured earlier (130.1 and 127.7 days) as well as having a lower height (109.5 and 102.3 cm) in Year 2 when compared to Year 1, respectively. Across recombination cycles, GS on average shortened the growing cycle of the selected lines. In Year 2, no significant differences were observed between recombination cycles. Across both years combined, DTH and DTM decreased by 2.8 and 1.5 days with respect to cycle C_0_, including the subset of the initial parents. During the first recombination cycle, GS produced significantly taller plants (on average 3.7 cm from C_0_ to C_1_), while in the subsequent cycles, PH slightly increased, but with no significant change. For all 3 traits, C_0_ showed nonsignificant values compared to the check.

### Genetic diversity was maintained in each of the selection cycles

**Fig. 3.**
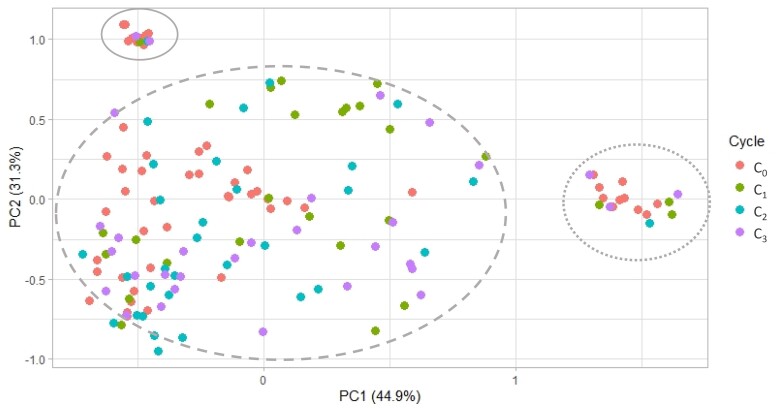
Principal component analysis based on Nei’s standard distance including all parents in C_0_ and F_6_ lines derived from selection cycles C_1_, C_2_, and C_3_. Circles display distinct groups, group 1 (solid line), group 2 (dashed line), and group 3 (round dotted line).

The genetic diversity in each of the selection cycles computed by Nei’s standard genetic distance is displayed in Table 3. The overall mean genetic distances within and between cycles were very similar. Mean genetic distances between the initial parents in C_0_ were higher than between the F_6_ lines in each recombination cycle, but mean distances between the F_6_ lines did not significantly decline from cycle C_1_ to C_3_. The largest genetic distance was observed between the group of C_0_ parents and the F_6_ lines in C_3_. Principal component analysis at a 2-dimensional scale depicted 3 groups for the initial 60 parents (Fig. 3). The 2 smaller groups (groups 1 and 3) each comprised the 10 selected parents of one F_4_ family (families 2 and 12). The larger group (group 2) included the parents of the 4 additional F_4_ families (families 3, 4, 11, and 13) characterized by their common parent “KACHU.” Lines in each of the selection cycles follow approximately the same, but wider patterns driven by intercrossing.

**Table 3.** Mean and standard deviation of Nei’s standard genetic distance within and between lines of each selection cycle.

			Mean distance between cycles		
Cycle	No. of lines	Mean distance within cycle	C0	C1	C2
C_0_	60	0.352 (±0.13)			
C_1_	29	0.327 (±0.07)	0.344 (±0.09)		
C_2_	32	0.320 (±0.07)	0.345 (±0.08)	0.330 (±0.07)	
C_3_	31	0.333 (±0.07)	0.354 (±0.10)	0.332 (±0.08)	0.328 (±0.07)

## Discussion

Accelerating the genetic progress of major cultivated crops such as wheat, maize, and rice is necessary to increase production in response to the global food crisis (Bentley *et al.* 2022). In autogamous crops, bulk and pedigree methods of breeding, which are based on inbred line selection, are commonly used in genetic improvement programs. These methods, however, produce limited novel combinations of genes in a breeding population. Recurrent selection promotes recombination and produces novel combinations of genes in a breeding population, but it requires accurate single-plant evaluation. GS, which can predict the breeding value of individuals based on their marker genotype, provides the potential to give a higher reliability of single-plant evaluations and to, therefore, be effective in recurrent selection. In this study, we implemented RCRGS in bi-parental spring wheat populations of CIMMYT spring bread wheat to evaluate its feasibility and estimate potential realized genetic gain. RCRGS was applied for GY in recombined F_1_s that originated from 14 CIMMYT crosses, based on 16 elite breeding lines.

Our results showed a consistent genetic gain for GY when summarized across 2 years of phenotyping. Genetic gain varied in percentage per cycle and in individual years and was not significant from C_1_ to C_2_. The highest gain was revealed from C_0_ to C_1_ and the lowest from C_1_ to C_2_. For the 2 years of phenotyping combined, genetic gain reached 12.3% from C_0_ to C_3_ and realized gain was 0.28 ton ha^−1^ per cycle. This genetic gain was slightly higher than expected from the GEBVs reported in each selection cycle, with an estimated realized genetic gain of 0.26 ton ha^−1^. These differences in genetic gain are anticipated and might be explained by additional G × E interactions during field evaluation, as well as other factors for example the choice of the prediction model and the genotyping platform. GEBVs indicated a slight decline for GY from C_1_ to C_2_ for the F_1_ individuals that were selected as parents and advanced to F_6_. However, this decline was not apparent for the observed GY in field evaluations in Year 1 and across the 2 years combined, while in Year 2, GY was lower in C_1_ and C_2_.

To further compute the realized genetic gain per year (ton ha^−1^ year^−1^), it is necessary to account for the number of cycles per year (2–3 cycles per year in this study) and for the time from the initial cross to the last cycle (theoretically 3.5 years from F_1_ development to the harvest of the C_3_F_6_ lines in this study, but extended to 5 years as 2 cycles were repeated due to logistical constraints). Therefore, given that GY from C_0_ (6.88 ton ha^−1^) to C_3_ (7.73 ton ha^−1^) increased by 12.3%, the average genetic gain of 0.28 t/ha per cycle is equivalent to 0.187 ton ha^−1^ year^−1^ [i.e. (3 · 0.28)/5] under our conditions and equivalent to 0.242 ton ha^−1^ year^−1^ under optimal theoretical conditions. Crespo-Herrera *et al.* (2017) analyzed genetic gain in CIMMYT Elite Spring Wheat Yield Trial in a period of 8 years from 2006–2007 to 2014–2015 and across 426 international locations classified in 3 target populations of environments. The highest genetic gain reached 0.102 ton ha^−1^ year^−1^ in optimally irrigated environments relative to a widely grown cultivar “ATTILA” and 0.044 ton ha^−1^ year^−1^ relative to several local checks. Mondal *et al.* (2020) reported the grain yield progress in CIMMYT spring bread wheat over 50 years determined in field trials during 5 crop seasons performed at CENEB under simulated field conditions. The highest genetic gains per year accounted for 0.035 and 0.031 ton ha^−1^ year^−1^ under irrigated and rainfed (limited drip irrigation) conditions, respectively. Therefore, the short-term genetic gain from RCRGS observed in the populations used in this study (0.187 ton ha^−1^ year^−1^) is higher (up to six times) than observed in previous CIMMYT studies under phenotypic selection, which, however, were longer-term studies between 8 and 50 years and were achieved in national trials in the case of Crespo-Herrera *et al.* (2017). The results we obtained reinforce the results by Bonnett *et al.* (2022) and highlight the potential of GS-assisted recombination at early breeding generations for achieving high genetic gain for GY.

Our study presents the second empirical report of RCRGS in wheat and the first for a complex trait such as GY. In a previous study, Veenstra *et al.* (2020) reported the improvement in nutritional quality of wheat via recurrent GS. The authors determined the realized genetic gain from GS for wheat grain fructan content by applying truncated selection (TS) and optimized contribution selection (OCS). GS led to a 25 ± 12% and 24 ± 6.4% increase in wheat grain fructans using TS and OCS, respectively. OCS showed a simultaneously greater retention of genetic variance and lower inbreeding levels.

High rates of inbreeding per breeding cycle with GS have been observed in simulations and empirical studies (Jannink *et al*. 2010; Rutkoski *et al.* 2015; Lin *et al.* 2017; Gorjanc *et al.* 2018). Rutkoski *et al.* (2015) found significant increases in inbreeding after 1 and 2 cycles of GS when compared with C_0_, significantly greater than the expected value under random genetic drift for all populations. Several methods to control the rate of inbreeding have, therefore, been proposed, including OCS (Meuwissen 1997) or optimal cross-selection (Gorjanc *et al.* 2018), in the population improvement context to improve selection and crossing plans. In this study, we only used TS and did not specifically consider maintaining genetic diversity in our crossing plans. Genetic diversity declined comparing cycle C_0_ with the subsequent recombination cycles but was well maintained from cycle C_1_ to C_3_. Thus, our results only partially agree with the findings reported in earlier studies with a reduced genetic variation. Zhang *et al.* (2017) reported similar results deploying rapid-cycle GS in multi-parental tropical maize populations, with a slightly narrowed genetic diversity only during the last GS cycles (C_3_ and C_4_). In this study, we balanced crosses within and between bi-parental F1s for each recombination cycle, generating 3 times more crosses between bi-parental F1s than within F1s, which we propose to be a reason that genetic diversity remained at a similar level throughout recombination cycles.

RCRGS was associated with a change in agronomic traits that were measured (DTH, DTM, and PH) in our study. Lines in cycle C_3_ had a shorter crop cycle, and lines in cycles C_1_ to C_3_ were taller. We, therefore, suggest that selection should be applied through a selection index to optimize selection of multiple traits. Each additional trait added to a selection index usually takes away some of the selective pressure that can be applied to other traits. Furthermore, tradeoffs exist between progress in one trait versus others. Nonetheless, several recently published studies show an increase in the prediction accuracy of genomic multi-trait selection over genomic single-trait selection (Montesinos-López *et al.* 2016, 2019). It will be important to further investigate multi-trait selection to optimize the predictive power of RCRGS.

We calculated GEBVs based on the nonlinear Gaussian kernel function, including the relationship matrix ***A*** (P + RKHS-KA) for the selection of new parents in each cycle. We favored this model because it demonstrated a significant positive correlation between observed yields of F_2:4_ lines and predicted GEBVs of F_2_ single plants in the study of Bonnett *et al.* (2022). The predictions of F_2_s in Bonnett *et al.* (2022) derived from crosses between inbreds that were part of the training population showed very little to no correlation between models. In contrast, the correlation of GEBVs derived from different models in this study was positive and still moderate after 3 cycles of recombination. Models including the pedigree information predicted higher yields and genetic gain calculated from the GEBVs in each selection cycle. These were closer to the observed realized genetic gain than models only using the marker information, which underlines our previous results that when molecular markers and pedigree information are considered jointly, prediction abilities are slightly but consistently superior to the marker or pedigree-derived models alone (de los Campos *et al.* 2009; Crossa *et al.* 2010).

Rapid generation advance or speed breeding can achieve up to 6 generations by year for spring wheat (Watson *et al.* 2018) with adequate infrastructure and trained staff in place. In this study, we achieved 3 to 4 crop cycles per year by taking several practical considerations in the RCRGS scheme into account. A very fast crop cycle provides only a short time from planting to flowering. In an RCRGS breeding scheme, breeding teams need to acquire DNA from seedling tissue, receive genotypic data, and run the statistical models to make parental predictions prior to the plants reaching the flowering stage, demonstrating a logistical challenge that requires careful planning and good communication within the team and with external genotyping providers, which are regularly used in public breeding programs. In addition, for repeated crossing in recombination cycles, male and female parents need to be sown at 2 to 3 different dates, to match the flowering of the selected parents. This extends the length of the greenhouse cycle, and some crosses might fail, making a full standardization of the scheme (with a constant number of crosses and offspring) difficult. Also, greenhouse-grown plants in pots are usually smaller and produce less seed. For the 3 recombination cycles in this study, the seed of the F_1_ individuals was limited in several cases. Being potential new parents, F_1_s were sown at 3 dates for subsequent crossing, and insufficient seed remained for selfing. It could, therefore, be recommended to apply GS at the F_2_ or F_3_ breeding generation to bypass the limited amount of seed for selfing (Gorjanc *et al.* 2018). We performed 3 recurrent GS cycles and evaluated derived lines at the end of the experiment. In a 2-part strategy as suggested by Gaynor *et al.* (2017), selected plants should be advanced directly for product development. This implies that recurrent cycles in the greenhouse must be aligned to the crop cycles of product development in the field. Overall, these and other logistical constraints remain a barrier to the practical application and implementation of RCRGS for many breeding programs.

## Conclusions

Different GS strategies are likely to be relevant in individual breeding programs and each program must determine which strategy is the best choice. This will be specific to the biological specificities of a crop, the breeding organization itself, and its economic context. Wheat breeding programs tend to use GS to control for G × E interaction, predicting the total genetic values of individuals, where both additive and nonadditive effects determine the final commercial value of the lines. Practical implementation of rapid-cycling GS strategies in wheat is still lacking and our study shows the potential of RCRGS to increase genetic gains for GY. Further work is needed to evaluate and optimize these GS strategies in wheat and other crop species in order to support the acceleration of current breeding progress.

## Supplementary Material

jkad025_Supplementary_Data

## Data Availability

The genotypic and phenotypic data of the training population and final F_6_ lines underlying this article are available in the CIMMYT data repository Dataverse (https://hdl.handle.net/11529/10548816). Supplemental material available at G3 online.
